# Integrated Metabolomics and Flavor Profiling Provide Insights into the Metabolic Basis of Flavor and Nutritional Composition Differences Between Sunflower Varieties SH363 and SH361

**DOI:** 10.3390/foods15010106

**Published:** 2025-12-30

**Authors:** Yanli Li, Huihui Gong, Xinxiao Cui, Xin Wang, Ying Chen, Huiying Li, Junsheng Zhao

**Affiliations:** 1 Institute of Industrial Crops, Shandong Academy of Agricultural Sciences, Jinan 250100, China; 2 Sunflower Research Institute, Baicheng Academy of Agricultural Sciences, Baicheng 137000, China

**Keywords:** sunflower, metabolomics, sensory quality, flavor, nutritional composition

## Abstract

Sunflower (*Helianthus annuus* L.) seeds exhibit variation in flavor and nutritional quality. In this study, we combined metabolomics (UPLC–MS/MS and GC–MS) with nutritional analysis and a database-driven flavoromics approach to elucidate the biochemical basis of quality differences between sunflower varieties SH361 and SH363. A total of 1448 seed metabolites were identified, with 242 varying between varieties (predominantly flavonoids and terpenoids). Based on the known aroma descriptors of identified metabolites, we inferred that SH363 would have a more intense nutty-aromatic flavor, whereas SH361 would be characterized by a predominantly sweet taste. SH363 seeds also contained ~50% oil (versus ~24% in SH361) and lower sugar content, indicating an inverse oil–sugar balance that is associated with more complex flavor notes. Lipids and aromatic terpenoids were identified as likely key contributors to SH363’s richer flavor profile. Overall, although limited to two genotypes, this work provides new insights into the metabolic basis of sunflower seed flavor differences and supports a conceptual model of lipid-associated flavor enhancement. These findings offer valuable guidance for breeding programs aimed at improving sunflower seed flavor and nutritional quality.

## 1. Introduction

Sunflower (*Helianthus annuus* L.) is a globally important oilseed crop, ranked as the world’s fourth largest source of edible vegetable oil and contributing to roughly 12% of global edible oil production [1,2]. The crop’s versatility—in providing oil, protein-rich meal, and edible seeds—has driven its widespread cultivation across temperate regions of the Americas, Europe, Asia, and Africa. Major producers include Ukraine, Russia, Argentina, China, Romania, and Turkey [3]. Annual worldwide sunflower seed output is on the order of tens of millions of tons (approximately 50 million metric tons in recent years) [1,4]. Sunflower oil, together with palm, soybean, and rapeseed oils, constitutes a core component of the global edible oil supply. The continued expansion of sunflower cultivation reflects sustained demand for its products.

Historically, breeding efforts in sunflower have prioritized agronomic traits like high seed yield and disease resistance, often at the expense of seed quality attributes such as flavor and nutrition. For example, in commercial confectionery sunflower hybrids, SH361 is favored by growers for its high yield (accounting for ~45% of planting area in 2018), whereas SH363—though planted on a smaller scale—commands a premium price due to its superior taste (noted for a uniquely crispy, nutty flavor) [5]. Despite advances, the biochemical basis for these flavor differences remains poorly understood, and identifying the key metabolic compounds underlying sunflower seed flavor is an ongoing research goal [6]. Improved knowledge in this area is needed to guide breeding strategies that enhance seed taste and aroma.

Sunflower seed quality depends not only on agronomic traits but also on the nutritional and physicochemical composition of the kernels. Key macronutrients–sugars, lipids, and proteins–greatly influence taste and aroma. For example, sugars evoke sweetness [6] and serve as substrates in Maillard browning, generating numerous flavor compounds [7]. Amino acids (from protein) provide umami taste and Strecker precursors for savory volatiles [8]. Equally important, sunflower kernels are inherently oil-rich–USDA data report ~51% fat on a dry basis (other analyses cite up to ~65%) [6,9]. This high lipid content underlies the characteristic nutty, roasted aroma of sunflower; fats carry oil-soluble aroma precursors and affect mouthfeel. In contrast, structural carbohydrates are minor: sunflower seed starch is very low (~0.4%) [10], and most fiber is cellulose in the hull (≈50% of hull weight), mainly influencing texture [11].

Varieties SH363 and SH361 have emerged as popular choices in the commercial sunflower seed market, yet they display distinct quality characteristics. SH363 is highly regarded for its superior taste, characterized by a more intense and balanced flavor profile, while SH361 lags behind in sensory appeal. These quality disparities impact consumer preferences and have economic implications for farmers, processors, and traders [5]. However, the metabolic mechanisms governing the formation of these flavor and quality traits remain unclear. Previous research has predominantly focused on individual metabolite groups or specific biochemical pathways, providing only fragmented insights. For instance, one study examined how fatty acid composition determines sunflower seed oil quality [12], and a survey of diverse sunflower germplasm revealed wide variation in seed oil fatty acid profiles (oleic acid ranging from ~19% to 79%, linoleic acid ~10% to 52%), with high-oleic cultivars exhibiting improved oil stability and nutritional value [1]. Other work has explored the influence of amino acids on the taste and nutritional quality of sunflower seeds [13]. However, these studies provide only a partial understanding of the complex metabolic networks involved in sunflower seed quality formation.

The intricate nature of sunflower seed quality, which results from the interplay of multiple metabolic pathways and the cumulative effects of numerous metabolites, necessitates a comprehensive systems-level approach [14]. Metabolomics, a powerful analytical technique that enables the simultaneous identification and quantification of a large number of metabolites in a biological sample, offers a unique opportunity to gain a holistic view of the metabolic landscape of sunflower seeds [12]. Recent advancements in metabolomics technologies, such as ultra–performance liquid chromatography–tandem mass spectrometry (UPLC–MS/MS) and gas chromatography–mass spectrometry (GC–MS), have significantly improved the sensitivity, specificity, and throughput of metabolite analysis [15,16]. These techniques can detect a wide range of metabolites, including primary metabolites (carbohydrates, amino acids, and organic acids) as well as secondary metabolites (flavonoids, terpenoids, phenolics, etc.), thus providing valuable insights into the metabolic pathways underlying sunflower seed quality [17].

In this study, we performed an integrated metabolomic comparison of SH363 and SH361 sunflower seeds using both UPLC–MS/MS and GC–MS platforms. This was coupled with targeted compositional analyses of key nutritional components (sugars, lipids, proteins, and others). Our goal was to elucidate the metabolic basis of the quality differences between these two varieties by identifying differentially accumulated metabolites and the pathways in which they are involved. We also examined how the contrasting nutritional profiles of SH361 (high sugar, low fat) and SH363 (low sugar, high fat) relate to their flavor and aroma characteristics. By linking chemical profiles to flavor attributes, this work establishes a foundation for targeted breeding strategies and quality improvement programs. Our findings are presented as testable hypotheses for sunflower flavor improvement, with the ultimate aim of developing high-quality sunflower varieties to meet consumer and market needs.

## 2. Materials and Methods

### 2.1. Experimental Materials

Sunflower varieties SH361 and SH363 seeds were sourced from Sanrui Company of Inner Mongolia. In April 2023, these seeds were planted in a field located in Linqing under uniform agronomic conditions. The experimental field was carefully selected to ensure homogeneous soil conditions, with soil samples analyzed prior to planting to determine its physical and chemical properties (pH, organic matter content, and nutrient levels). Standard agricultural practices (uniform irrigation, fertilization, and pest control) were strictly followed throughout the growth cycle to minimize environmental variations. The seeds were harvested at full maturity following the standard protocol for sunflower, and were immediately stored under controlled conditions at 10 °C and 55% relative humidity to maintain seed quality until further analysis [18]. Each variety’s harvested seed lot was divided into three independent sample replicates for analysis.

### 2.2. Sample Preparation

#### 2.2.1. LC-MS Sample Preparation

For the liquid chromatography–mass spectrometry (LC–MS) analysis, biological samples underwent a series of meticulous steps. Firstly, the sunflower seed samples were placed in a Scientz–100F freeze–dryer. The freeze–drying process was carried out under vacuum conditions at a temperature of −50 °C for 48 h to completely remove moisture while preserving the integrity of the metabolites. Subsequently, the dried samples were transferred to a MM 400 grinder (Retsch, Haan, Germany) and ground into a fine powder at a frequency of 30 Hz for 1.5 min to ensure uniform particle size.

An Electronic balance (MS105DΜ, Mettler Toledo, Greifensee, Switzerland) with a precision of 0.01 mg was used to accurately weigh 50 mg of the sample powder into a 2 mL centrifuge tube. Then, 1200 μL of pre–cooled (−20 °C) 70% methanol water solution containing a known concentration of internal standard was added to the tube. The mixture was vortexed for 30 s every 30 min, for a total of 6 times, to ensure thorough extraction of metabolites. After extraction, the samples were centrifuged at 12,000 rpm for 15 min at 4 °C. The supernatant was carefully collected and filtered through a 0.22 μm pore size microporous membrane to remove any particulate matter. The filtered supernatant was then transferred to an injection vial for UPLC–MS/MS analysis [19].

#### 2.2.2. GC-MS Sample Preparation

For gas chromatography–mass spectrometry (GC–MS) analysis, the samples were retrieved from a −80 °C freezer. To prevent metabolite degradation due to temperature changes, the samples were immediately ground in liquid nitrogen using a mortar and pestle. After thorough grinding, approximately 0.2 g of each sample was accurately weighed into a 20 mL headspace vial. Subsequently, 0.2 g of NaCl powder was added to each vial to enhance the extraction efficiency of volatile compounds, and 20 μL of a 10 μg/mL internal standard solution was introduced into the vial.

Volatile extraction was performed using an automated headspace solid-phase microextraction (HS-SPME) device. The extraction parameters were optimized as follows: each vial was heated to 60 °C for 30 min to allow volatile compounds to partition into the gas phase, and then an SPME fiber (50/30 μm divinylbenzene/carboxen/polydimethylsiloxane,) was exposed to the headspace for 40 min at 60 °C to adsorb the volatile metabolites. After extraction, the fiber was immediately transferred to the GC–MS system for analysis [20].

### 2.3. Chromatography–Mass Spectrometry Conditions

#### 2.3.1. UPLC–MS/MS Conditions

The UPLC separation was carried out on an ExionLC™ AD system (SCIEX, Framingham, MA, USA) using a Waters ACQUITY UPLC HSS T3 C18 column (2.1 mm × 100 mm, 1.8 μm particle size). The mobile phase consisted of solvent A (0.1% formic acid in water) and solvent B (0.1% formic acid in acetonitrile). The gradient elution program was as follows: 0–1 min, 5% B; 1–9 min, 5–95% B; 9–10 min, 95% B; 10–10.1 min, 95–5% B; 10.1–12 min, 5% B (re-equilibration). The flow rate was set at 0.3 mL/min, and the column temperature was maintained at 40 °C. The injection volume was 2 μL.

The MS/MS detection was performed on a TripleTOF 6600 mass spectrometer (SCIEX) with electrospray ionization in both positive and negative ion modes. The ESI source parameters were optimized as follows: ion spray voltage, +5500 V (positive)/−4500 V (negative); curtain gas, 30 psi; nebulizer gas, 50 psi; heater gas, 55 psi; source temperature, 550 °C. The mass range was set from m/z 50–1200, and the total acquisition time per sample was 12 min. Multiple reaction monitoring (MRM) mode was used for quantitative analysis, with collision energy and declustering potential optimized for each target metabolite [21,22].

#### 2.3.2. GC–MS Conditions

The GC–MS analysis was conducted on an Agilent 7890B gas chromatograph coupled with an Agilent 5977A mass spectrometer. Chromatographic separation was achieved using a DB–5MS capillary column (30 m × 0.25 mm × 0.25 μm). Helium was used as the carrier gas at a constant flow rate of 1.0 mL/min. The temperature program was: initial 40 °C (held for 2 min), then ramped at 5 °C/min to 280 °C and held for 10 min. The split ratio was set at 1:10, and the injection volume was 1 μL.

The mass spectrometer was operated in electron ionization (EI) mode at 70 eV. The ion source temperature was 230 °C, and the quadrupole temperature was 150 °C. The mass range was scanned from m/z 35–550, and the scan time was 0.2 s. Compound identification was performed by comparing the mass spectra with the NIST 2014 mass spectral library and by calculating retention indices for confirmation against reference values [23].

### 2.4. Nutritional Component Analysis

Three biological replicate analyses were performed on sunflower seeds of varieties SH361 and SH363. Finely ground seed powder was used for all assays.

#### 2.4.1. Total Sugars

Defatted seed powder (~0.05 g) was extracted in hot water, and the extract was subjected to acid hydrolysis to convert all carbohydrates (soluble sugars and starch) into reducing sugars. The resulting solution’s reducing-sugar content was measured by the 3,5-dinitrosalicylic acid (DNS) colorimetric assay [24]. In this assay, reducing sugars reduce the yellow DNS reagent (under alkaline, high-temperature conditions) to 3-amino-5-nitrosalicylic acid, yielding an orange-red color whose intensity (absorbance at 540 nm) is proportional to the reducing sugar concentration. Each sample was mixed with DNS reagent and boiled for 10 min, then cooled and diluted. Absorbance at 540 nm was recorded, and a glucose standard curve was used to convert absorbance to milligrams of sugar per gram of sample (mg/g). This DNS method provides a measure of “total sugars” (reducing sugars plus those released from hydrolyzed starch) in the sample. A reference standard method for total carbohydrate analysis was followed. All measurements included appropriate blanks, and each assay was replicated three times. Results are reported as mean ± standard deviation.

#### 2.4.2. Crude Fat

Crude fat content was determined by the Soxhlet extraction method, following the procedure of the Chinese National Food Safety Standard GB 5009.6–2016 (First Method: Soxhlet) [25]. Approximately 5 g of dried seed powder was accurately weighed (to 0.001 g) and placed in a filter-paper thimble. The thimble was inserted into a Soxhlet extractor, and anhydrous petroleum ether (boiling range 40–60 °C) was used as the solvent. The solvent was heated to reflux (~6 cycles per hour) for about 6–8 h, during which lipids in the sample were continuously dissolved and extracted into the solvent. After the extraction period, the solvent in the receiving flask was distilled off (and recovered) until only the extracted oil remained. The flask containing the oil was then dried in an oven at 100 ± 5 °C for 1 h, cooled in a desiccator, and weighed. Drying and re-weighing to a constant weight ensured complete removal of solvent. The crude fat content was calculated as the mass of the extracted oil divided by the mass of the initial sample, expressed as grams of fat per 100 g of dry sample (percent by weight). This Soxhlet extraction procedure is an established standard for gravimetric fat determination and is recognized by AOAC as a reference method for crude fat analysis [26]. It exploits the high solubility of lipids in organic solvents to achieve exhaustive extraction of fats from the sample.

#### 2.4.3. Crude Protein

Crude protein was measured by the Kjeldahl nitrogen determination method, according to GB 5009.5–2016 (First Method: Kjeldahl) [27]. Approximately 0.1 g of finely ground seed powder was digested in concentrated H_2_SO_4_ along with catalyst salts (e.g., K_2_SO_4_ to raise boiling point, and a trace of CuSO_4_ or selenium as a catalyst) using a Kjeldahl digestion apparatus. During this digestion step, organic nitrogen in the sample is converted to ammonium sulfate. The digest mixture was heated until it became clear (bluish-green in color), indicating complete digestion, then it was cooled and diluted with distilled water. The solution was made strongly alkaline by adding NaOH, and a Kjeldahl steam distillation was performed to liberate ammonia from the ammonium sulfate. The released ammonia gas was captured into a receiving flask containing boric acid solution with mixed indicators (methyl red and bromocresol green). The amount of ammonia (which is proportional to the total nitrogen in the sample) was then quantified by titration with standardized HCl solution until the indicator endpoint (color change) was reached. A reagent blank (containing no sample) was carried through the entire procedure for correction. The crude protein content was calculated as total nitrogen (N) multiplied by 6.25, where 6.25 is the standard conversion factor from nitrogen to protein for plant materials (based on proteins being ~16% nitrogen). This Kjeldahl method is a classical standard for protein content determination and has been almost universally applied for protein analysis in foods [28]. Results are reported as % protein (g protein per 100 g dry sample). All samples were analyzed in triplicate for precision.

#### 2.4.4. Starch

Starch content was determined by an acid hydrolysis–anthrone colorimetric method. First, the sample’s starch was extracted and hydrolyzed to glucose. Defatted seed powder (~0.03–0.05 g, with sample size adjusted for high-starch materials) was pre-treated with 80% ethanol to remove soluble sugars, then the remaining insoluble residue (containing starch) was gelatinized in boiling water and subsequently hydrolyzed to glucose by adding concentrated HCl. After hydrolysis (under reflux in acid for a specified time), the mixture was neutralized and/or appropriately diluted. The glucose released from the starch was then quantified using the anthrone–sulfuric acid assay. In this assay, anthrone reagent (0.2% anthrone in concentrated H_2_SO_4_) is added to the sample solution, which is then heated in a boiling water bath for 10 min. Anthrone reacts with furfural derivatives produced from dehydrated glucose (and other sugars) to yield a blue-green colored complex. After the reaction, the tubes were cooled to room temperature and the absorbance of the solution was measured at 620 nm. A calibration curve was prepared using a series of glucose standard solutions, and the starch content of samples was calculated from the glucose-equivalent values. (A factor of 0.90 was applied to convert glucose to starch, to account for the water molecule removed when glucose units polymerize to form starch.) The anthrone method is a widely used colorimetric procedure for carbohydrate quantification and is suitable for measuring total starch after complete acid hydrolysis of the sample [29]. All samples were analyzed in triplicate, and results are expressed as mg starch per g dry sample.

#### 2.4.5. Cellulose

Cellulose content (as an estimate of crude fiber) was determined by an acid-detergent fiber (ADF) procedure, based on the standard Van Soest detergent fiber method [30] sequential extraction was performed to isolate the indigestible cell wall fiber fraction of the seed. Defatted seed powder (~0.02 g) was first boiled in 80% ethanol (Extraction Solution I) for 20 min to remove sugars and other solvent-soluble constituents. The residue was collected and washed sequentially with additional 80% ethanol and acetone (two washes each), yielding a crude fiber-rich precipitate. Next, to remove starch and proteins, the residue was incubated in a cold enzyme solution or detergent solution (Extraction Solution II) for ~15 h, then centrifuged and the solid residue was dried—this yielded the acid-detergent fiber (ADF) fraction, which consists primarily of cellulose and lignin. This fiber-rich ADF residue was then subjected to strong acid hydrolysis to solubilize the cellulose. Specifically, the dried ADF residue was resuspended in a small volume of water, and 72% H_2_SO_4_ (acid solution) was slowly added (0 °C, ice-bath) with stirring. The mixture was kept on ice for 30 min to allow the concentrated acid to break down (solubilize) the cellulose. After this, the mixture was diluted and centrifuged to stop the reaction. The supernatant, which contains glucose released from cellulose, was collected and analyzed by the anthrone–sulfuric acid colorimetric method (similar to the starch assay above). Anthrone reagent was added to the diluted supernatant, the mixture was heated at 95 °C for 10 min, then cooled, and the absorbance at 620 nm was measured. Glucose standards were used for calibration, and the cellulose content was calculated (with a 0.90 conversion factor from glucose to cellulose) and expressed as a percentage of dry weight. This acid-detergent fiber method is part of the Van Soest fiber analysis system, which provides a more accurate measure of plant cell-wall components than the traditional crude fiber assay. In the Van Soest system, neutral detergent fiber (NDF) includes hemicellulose, cellulose, and lignin, while ADF (acid detergent fiber) represents the fraction of cellulose + lignin. By subtracting lignin (determined as acid-detergent lignin) from ADF, one can estimate cellulose content. Our procedure effectively isolates and quantifies cellulose by removing other constituents and measuring the remaining fiber fraction’s carbohydrate content. All measurements were performed in triplicate and reported as mean values on a dry-weight percentage basis.

### 2.5. Sensory Attribute Analysis

A database-driven “flavoromics” strategy was used to infer sensory notes from the chemical data [31,32]. In this approach, each volatile metabolite detected in the seeds was annotated with its known odor/aroma descriptors as reported in public flavor databases. Specifically, each compound (e.g., terpenes, aldehydes, pyrazines) was cross-referenced against resources such as The Good Scents Company Information System, the Perflavory compound database, Leffingwell’s LRI & Odor database, and the Chinese Food Flavor Laboratory database. Ten core flavor categories (green, sweet, hay, dry, coffee-like, caramel-like, resinous, woody, pine, fresh) were defined based on the highest number of annotated metabolites, covering the dominant sensory traits of sunflower seeds. Additional extended descriptors (e.g., roasted, aromatic, nutty) were compiled in Table S2, encompassing all matched odor notes for comprehensive reference. Each volatile’s reported aroma descriptor was then mapped to one or more of these categories. The two seed varieties (SH361 vs. SH363) were compared qualitatively by examining which compounds (and their relative abundances) fell into each flavor category. This in silico profiling yields a predicted flavor profile difference between the varieties without a sensory panel. We emphasize that this method infers relative aroma attributes from chemistry rather than providing direct sensory scores. All statistical analyses were performed in R (v4.2.1) and GraphPad Prism 9, and nutrient values were compared by two-tailed t-tests (α = 0.05); data are reported as mean ± SD.

### 2.6. Data Analysis

For the LC–MS platform data, Analyst 1.6.3 software (SCIEX) was utilized for initial processing. The software performed peak detection based on signal–to–noise ratio and retention time window, followed by peak integration to calculate metabolite peak areas. Metabolite identification for LC–MS was achieved by matching the accurate mass and MS/MS fragmentation patterns of detected peaks with those in comprehensive metabolite databases such as the Human Metabolome Database (HMDB) and METLIN [33,34]. For the GC–MS data, Agilent MassHunter software was used for processing. Similarly, peak detection and integration were carried out, and metabolites were identified by comparing mass spectra to the NIST library. Additionally, retention indices for volatile compounds were computed and compared to reference values to improve identification accuracy [23].

Differential metabolites between SH361 and SH363 were screened using a combination of multivariate and univariate analyses. First, an orthogonal partial least–squares discriminant analysis (OPLS–DA) model was constructed to distinguish the two groups. The model was validated (Q^2^, R^2^Y, R^2^X and permutation tests) to ensure it was not overfitted. From the OPLS-DA, the variable importance in projection (VIP) for each metabolite was obtained as a measure of its contribution to group separation. Metabolites with VIP > 1 were considered influential. In parallel, independent two-sample t-tests were performed for each identified metabolite’s abundance between SH361 and SH363 to assess statistical significance. A threshold of *p* < 0.05 was used for significance in this exploratory analysis (no formal FDR correction was applied). Metabolites meeting both multivariate (VIP > 1) and univariate (|log_2_ fold-change| ≥ 1) criteria were designated as differential metabolites. These differential metabolites were annotated to KEGG pathways, and pathway enrichment analysis (Fisher’s exact test) was performed to determine which metabolic pathways were significantly overrepresented (*p* < 0.05) among the differential metabolites [35,36].

## 3. Results

### 3.1. Sample Quality Control

**Figure 1:**
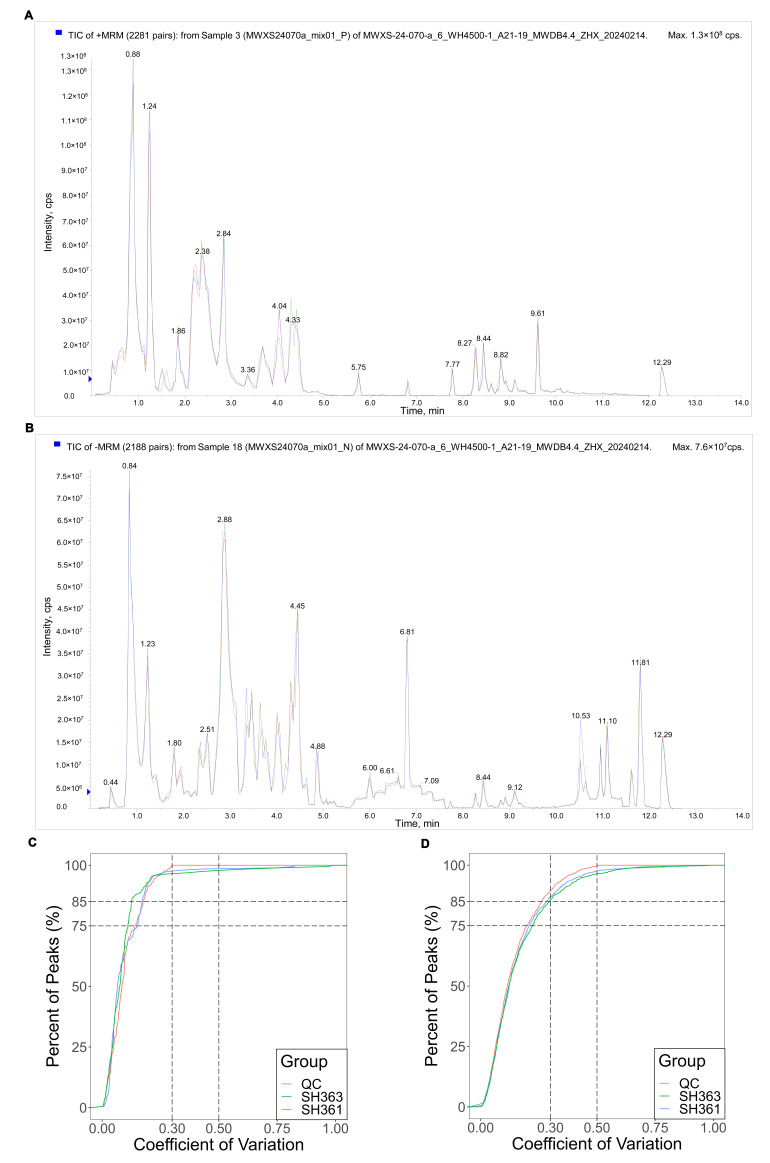
Quality control of metabolomic data for sunflower seed samples. (**A**) Overlaid TIC chromatograms in positive ion mode for three QC replicates (blue, red, green traces), showing nearly identical peak patterns across runs. (**B**) Overlaid TIC traces in negative ion mode for the QC replicates, likewise showing highly consistent profiles. (**C**) Empirical cumulative distribution function (ECDF) of coefficient of variation (CV) values for GC–MS peak areas, for QC (red curve) and sample replicates (green for SH363, blue for SH361). Vertical dashed lines mark CV thresholds at 0.3 and 0.5; horizontal dashed lines mark 75% and 85% cumulative percentage levels. Approximately 85% of QC peaks and 80% of sample peaks have CV < 0.3; by CV < 0.5, over 95% of peaks in all groups fall below the threshold. (**D**) ECDF of CV values for UPLC–MS peak areas, plotted with the same CV threshold lines (0.3 and 0.5) and reference levels (75%, 85%) as in (**C**). The QC curve is shifted toward lower CV values relative to the sample curves, indicating lower overall variability in the QC features (as expected for pooled quality controls).

Quality control (QC) samples (pooled from all test samples) were analyzed to evaluate data quality and instrument stability. The total ion current (TIC) chromatograms of QC samples from both UPLC-MS and GC-MS showed highly overlapping traces (Figure S1), indicating consistent retention times and peak intensities across runs. In both positive and negative ion modes for LC–MS, the TIC curves of three QC injections almost completely overlapped (Figure 1A,B), demonstrating excellent analytical repeatability. To assess signal stability, the coefficient of variation (CV) of metabolite peak areas was calculated for the QC replicates. Over 75% of detected features in QC had CV < 0.3 (Figure 1C,D), suggesting high precision and data reliability suitable for subsequent in-depth analysis [37].

### 3.2. Metabolite Profiling of Sunflower Seeds

**Figure 2:**
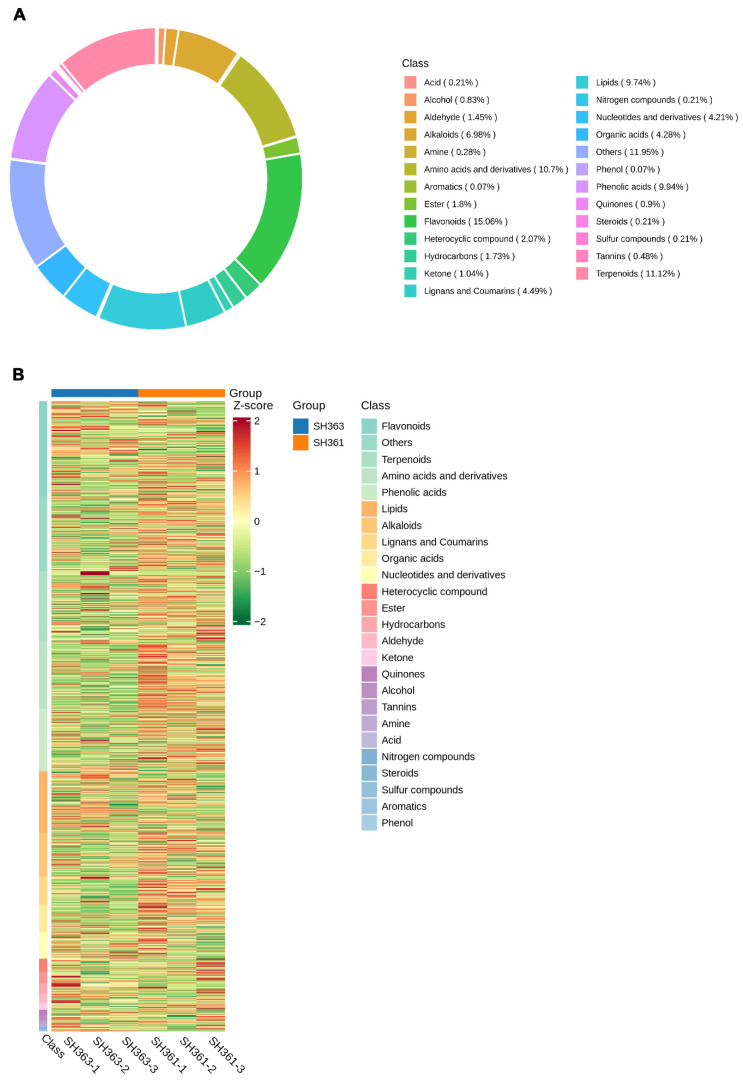
Overview of metabolite profiling results for SH363 and SH361 seeds. (**A**) Donut chart showing the class composition of all identified metabolites as percentages of the total metabolome. Each segment represents a metabolite class, with segment size proportional to that class’s contribution. Secondary metabolite classes collectively make up a large portion of the detected compounds in the seeds. (**B**) Heatmap of metabolite abundances (Z-score normalized) across all samples (three biological replicates each for SH363 and SH361). Each row represents one identified metabolite (rows are grouped by chemical class, indicated by a colored side bar), and each column represents a seed sample. Warmer colors (yellow) indicate higher relative abundance and cooler colors (blue) indicate lower relative abundance.

Using the UPLC–MS/MS and GC–MS dual-platform approach, we detected a total of 1448 metabolites in the sunflower seed samples. Among them, 365 were classified as primary metabolites, including 155 amino acids and derivatives (10.7%), 62 organic acids (4.28%), 61 nucleotides and derivatives (4.21%), 26 esters (1.8%), 21 aldehydes (1.45%), 15 ketones (1.04%), 12 alcohols (0.83%), 4 amines (0.28%), 3 steroids (0.21%), 3 acids (0.21%), and 3 nitrogen–containing compounds (0.21%). The remaining 910 were secondary metabolites, such as 218 flavonoids (15.06%), 161 terpenoids (11.12%), 144 phenolic acids (9.94%), 141 lipids (9.74%), 101 alkaloids (6.98%), 65 lignans and coumarins (4.49%), 30 heterocyclic compounds (2.07%), 25 hydrocarbons (1.73%), 13 quinones (0.9%), 7 tannins (0.48%), 3 sulfur–containing compounds (0.21%), 1 aromatic compound (0.07%), and 1 phenol (0.07%). The class distribution of all identified metabolites is presented in Figure 2A. This diverse metabolite profile reflects the complex chemical composition of sunflower seeds.

A cluster analysis (unsupervised hierarchical clustering) of metabolite abundance profiles was performed across all samples. The resulting heatmap (Figure 2B) showed that the metabolite profiles of biological replicates were very similar within each variety, and that the two varieties formed distinct clusters, indicating clear metabolic differences between SH363 and SH361. This distinct separation in metabolic profiles suggests that the chemical composition differences might underlie their observed quality differences [38].

### 3.3. Multivariate Statistical Analysis of Metabolites

**Figure 3:**
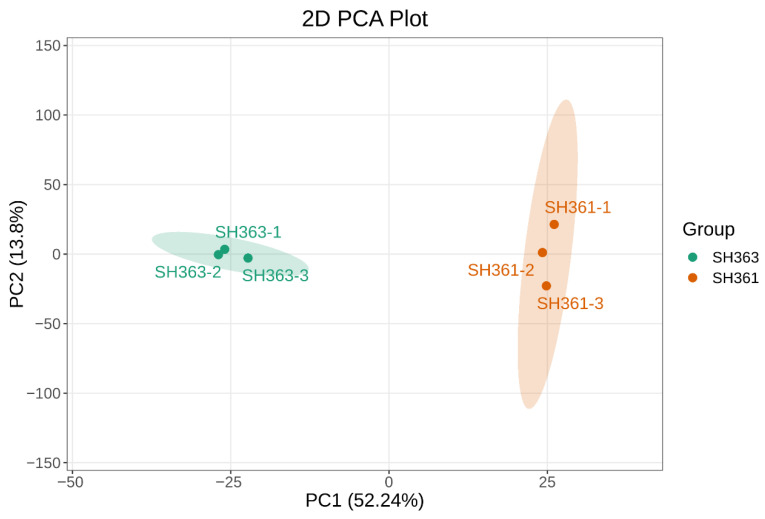
Principal component analysis (PCA) of sunflower seed metabolomes (SH363 vs. SH361). Score plot of the first two principal components (PC1 and PC2), which explain 52.24% and 13.8% of the variance, respectively. Each point represents an individual seed sample (n = 3 per variety), with SH363 samples in green and SH361 samples in orange. Ellipses indicate the 95% confidence regions for each variety (Hotelling’s T^2^). The PCA plot shows that SH363 and SH361 samples separate along PC1, while samples cluster tightly within each variety (illustrating the clustering of biological replicates).

Principal component analysis (PCA) was performed to assess the overall differences between the two sunflower varieties and the variation within each group. The PCA score plot (Figure 3) revealed that the samples of SH361 and SH363 had good within–group clustering, and the two varieties were clearly separated along the first principal component (PC1), which accounted for 52.24% of the variance. This result demonstrated that the metabolite accumulation patterns were significantly different between SH361 and SH363 [39].

**Figure 4:**
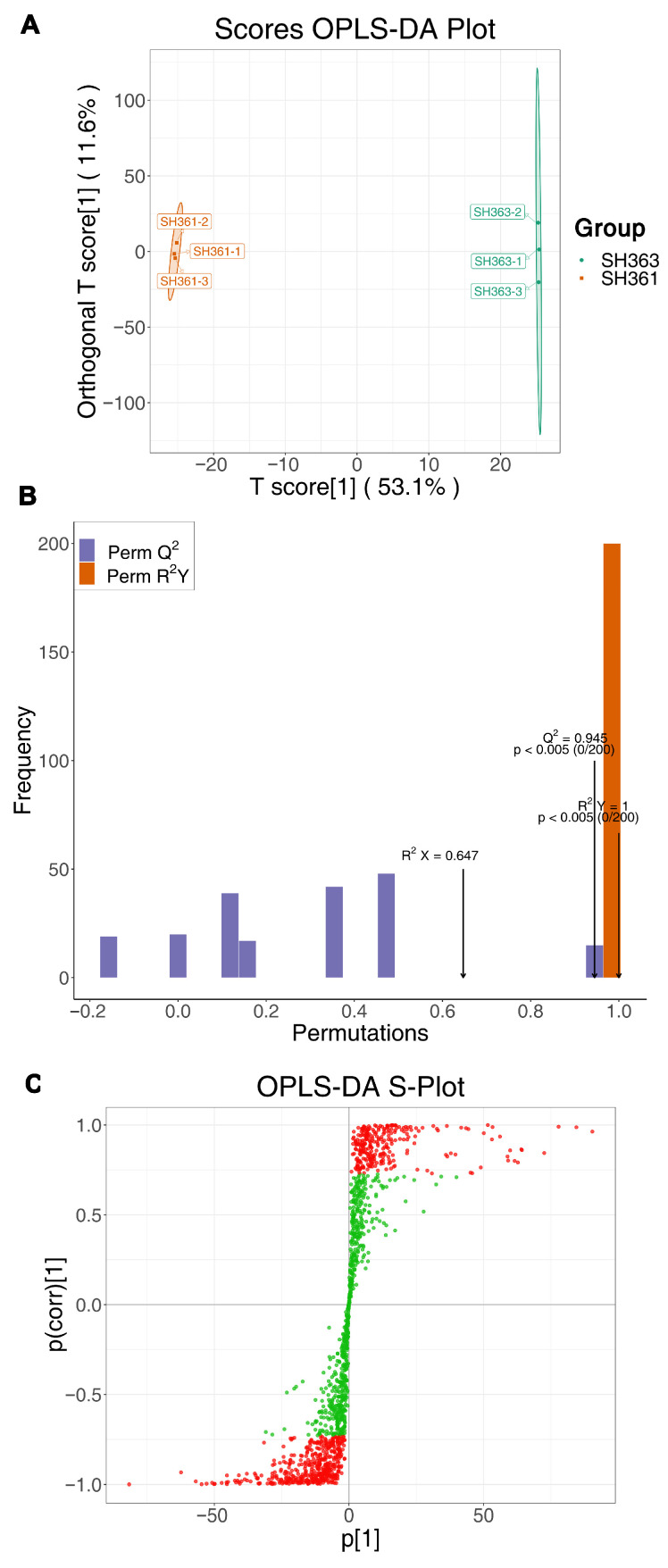
Multivariate analysis distinguishing SH363 and SH361 metabolite profiles. (**A**) OPLS-DA scores plot projecting samples onto the first predictive component (t[1], x-axis) and the first orthogonal component (orthogonal t[1], y-axis) of the model. Green circles: SH363; orange squares: SH361. The clear separation indicates distinct metabolomic signatures for the two varieties. (**B**) OPLS-DA model validation parameters. The bar chart shows R^2^Y (goodness of fit for class discrimination) and Q^2^ (predictive ability) for the model (red bars) versus distributions from 200 permutation tests (gray). The high original values and low permutation values (no overlap) confirm model validity (permutation test *p* < 0.005). (**C**) OPLS-DA S-plot identifying influential metabolites. Each point represents a metabolite, plotted by its covariance *p* (x-axis) and correlation *p*(corr) (y-axis). Metabolites farthest from the origin (in the top-right or bottom-left corners) have large and reliable differences between varieties.

To further interpret the metabolic differences, we applied multivariate statistical analyses. The OPLS-DA model effectively discriminated SH363 vs. SH361 metabolomes. Figure 4A showed a pronounced separation between SH361 and SH363 along the predictive component, with minimal within-group variation along the orthogonal component. Model validation metrics were excellent: as shown in Figure 4B, the model had Q^2^ = 0.955, R^2^Y = 1.0 (indicating high predictive ability and perfect class separation), R^2^X = 0.647 (fraction of metabolite variance captured by the model). Permutation tests yielded *p* < 0.005 for both Q^2^ and R^2^Y, confirming that the model is statistically robust and not overfitted. An S-plot from the OPLS-DA (Figure 4C) was used to identify the metabolites contributing most to the separation. In the S-plot, each point is a metabolite plotted by its covariance p (x-axis, reflecting magnitude of change between groups) and correlation p(corr) (y-axis, reflecting consistency of change across replicates). Metabolites at the plot extremes (high magnitude and high reliability) were considered important. We observed that many points on the right (high in SH363) correspond to terpenoids and lipids, whereas points on the left (high in SH361) included some sugar-related compounds. These high-[p], high-[p(corr)] features were deemed key discriminators driving the metabolic separation of SH363 and SH361 [40].

### 3.4. Differential Metabolites Between SH361 and SH363

**Figure 5:**
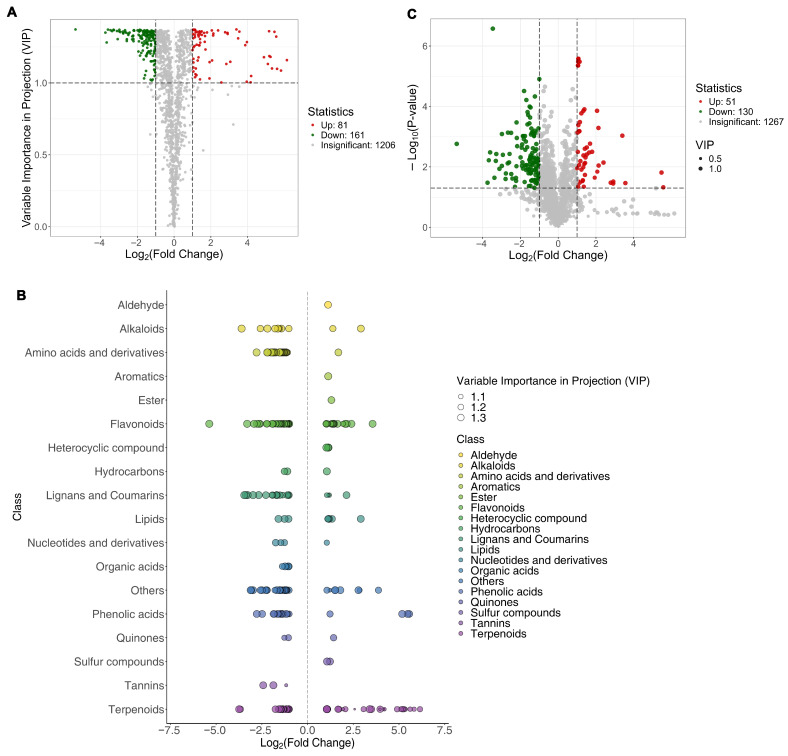
Metabolomic analysis reveals distinct chemical profiles between SH363 and SH361. (**A**) OPLS-DA volcano plot showing discriminating metabolites; red points indicate compounds more abundant in SH363, green points more abundant in SH361 (|log_2_FC| ≥ 1, VIP ≥ 1). (**B**) Chemical class distribution of differential metabolites grouped by compound family, with point size reflecting VIP scores. (**C**) Statistical volcano plot combining fold-change and significance testing; red and green points represent significantly different metabolites (*p* < 0.05, |log_2_FC| ≥ 1), gray points are non-significant.

Based on the OPLS-DA variable importance (VIP) scores and univariate fold-changes, we identified 242 metabolites that differed significantly between SH361 and SH363 (VIP > 1 and |log_2_FC| ≥ 1). Among these, 81 metabolites showed higher abundance in SH363 (up-regulated in SH363 or down in SH361) and 161 were higher in SH361 (down-regulated in SH363). These 242 differential metabolites span 18 chemical categories, with flavonoids (53 metabolites) and terpenoids (45 metabolites) being the largest groups. Within flavonoids, 21 were more abundant in SH363 and 32 higher in SH361; for terpenoids, 25 were higher in SH363 and 20 in SH361. Figure 5A shows a volcano plot of these differences, and the breakdown of differential metabolites by category is shown in Figure 5B.

Additionally, a volcano plot integrating fold-change and statistical significance (*p*-value) with VIP scores highlights the most significant metabolite differences between the varieties (Figure 5C). In this plot, metabolites exceeding both significance (*p* < 0.05) and fold-change (|log_2_FC| ≥ 1) thresholds are color-coded (red for higher in SH363, green for higher in SH361), with point size proportional to VIP score to emphasize multivariate importance. This integrated view underscores the key metabolite changes distinguishing SH363 from SH361.

### 3.5. Differential Metabolic Pathways Underlying Flavor Differences

**Figure 6:**
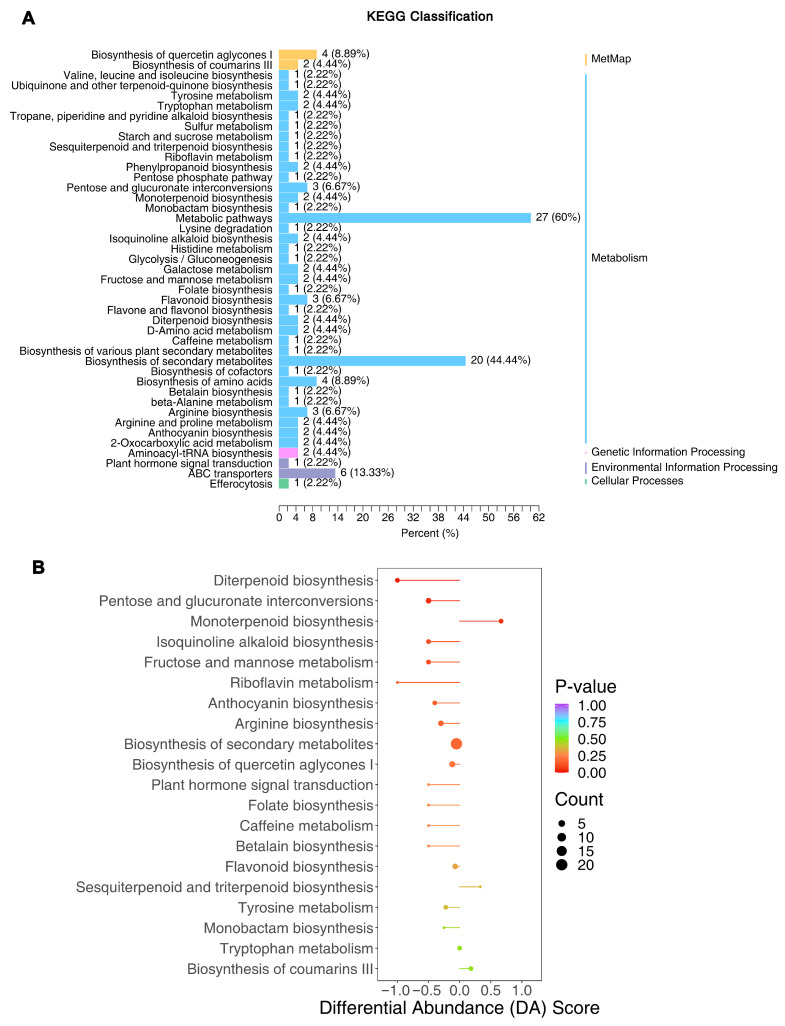
Pathway analysis of differential metabolites. (**A**) KEGG pathway classification of the 242 differential metabolites. Horizontal bars (color-coded by broad KEGG category) show the percentage of these metabolites associated with each metabolic pathway. Bar length corresponds to that percentage, with annotations of metabolite count and percentage of total differential metabolites. (**B**) KEGG pathway enrichment plot showing the differential abundance (DA) score for each pathway. Each point represents one pathway (*p* < 0.05). The x-axis is the DA score, reflecting the overall difference in metabolite levels between SH363 and SH361 (negative values indicate the pathway’s metabolites are, on average, more abundant in SH361; positive values indicate they are more abundant in SH363). Each point is plotted with a horizontal line representing the range (min to max) of individual metabolite contributions to that pathway’s score, and a central dot for the mean DA score. Point color reflects enrichment significance (blue for *p* ≈ 1, red for *p* ≈ 0) and size is proportional to the number of differential metabolites in the pathway.

Mapping the 242 differential metabolites to KEGG pathways showed that they were associated with a wide array of metabolic processes. In total, 118 of the differential metabolites could be assigned to 43 KEGG pathways. Figure 6A illustrates the pathways containing the most differential metabolites. Notably, the pathway “Biosynthesis of secondary metabolites” (a broad category) contained 20 of the differential metabolites (~44% of that subset), and general “Metabolic pathways” included 27 metabolites (~60%). This confirms that secondary metabolism, in particular, is a major area of divergence between SH361 and SH363. More specific pathway analysis (Figure 6B) identified two pathways significantly enriched in differential metabolites (Fisher’s exact test, *p* < 0.05 after Bonferroni correction): diterpenoid biosynthesis and pentose and glucuronate interconversions. The enrichment of these pathways suggests they play a role in the varietal differences.

Importantly, these particular pathways are linked to flavor relevant compounds. In the diterpenoid biosynthesis pathway, multiple terpenoid intermediates were higher in SH363 (Table S1); this corresponds with SH363’s greater abundance of aroma-contributing terpenoids and could explain its stronger “resinous” and “pine” aroma notes inferred from the metabolite profile. In turn, the pentose and glucuronate interconversion pathway was enriched due to significantly elevated sugar alcohols (xylitol, D-arabinitol, ribitol) in SH361 (Table S1). These sugar alcohols are products of carbohydrate metabolism (often accumulating via the pentose phosphate pathway) and were up-regulated in SH361 consistent with this variety’s higher sugar accumulation [41]. However, despite the higher flux through sugar-related pathways in SH361, SH363 demonstrated a more complex flavor profile and better nutritional balance, implying that factors beyond pentose metabolism (such as increased lipid and volatile synthesis in SH363) are key to its quality advantage. The accumulation of sugar alcohols in SH361 may serve osmoprotective or storage functions, reflecting a distinct metabolic strategy compared to SH363, but one that does not necessarily translate to superior flavor.

### 3.6. Nutrient Composition Differences

Basic compositional analysis of the seeds marked differences in macronutrient levels between SH361 and SH363 (Table 1). On a dry-weight basis, SH361 seeds contained substantially more total sugars but much less fat than SH363 seeds. Specifically, SH361 averaged ~32% total sugars, compared to ~25% in SH363, whereas SH361’s crude fat was only ~23.8%, versus ~51.0% in SH363. These differences in sugar and oil content were statistically significant (*p* < 0.01). Crude protein content was similar in both varieties (~27% in SH361 vs. ~26% in SH363, *p* > 0.05). Starch and cellulose levels were low (on the order of a few percent) and did not differ appreciably between the two varieties (each ~2–4%, differences not significant given the high variance). Thus, the most pronounced compositional difference is an inverse relationship between sugar and oil content: SH361 is a high-sugar/low-oil variety, while SH363 is a low-sugar/high-oil variety. This inverse sugar–lipid balance likely has implications for flavor, as discussed below.

### 3.7. Inferred Sensory Characteristics of Sunflower Seeds

**Figure 7:**
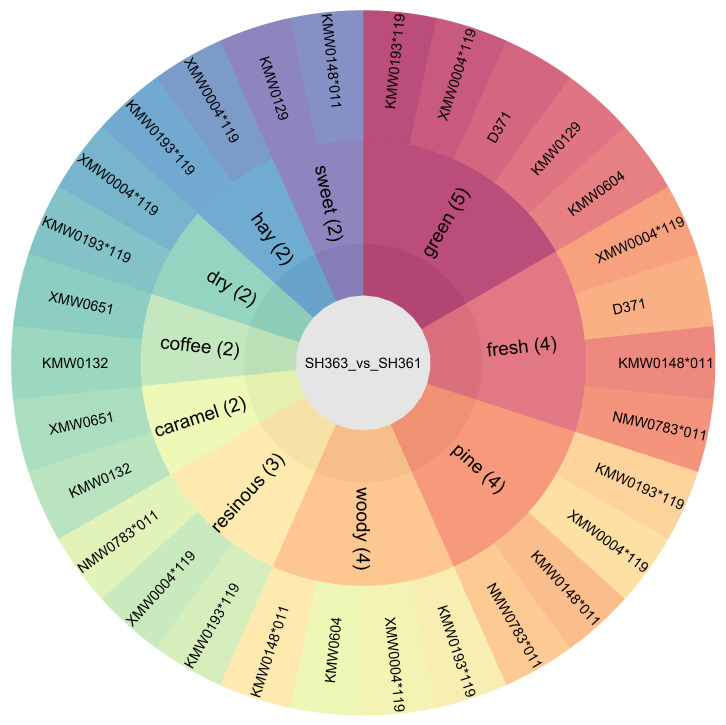
Sunburst plot linking aroma descriptor categories to their associated differential metabolites for SH363 vs. SH361. The inner ring shows 10 core aroma categories, with each segment labeled by the descriptor name and the number of differential metabolites (meeting the screening criteria: VIP > 1 and |log_2_FC| ≥ 1) annotated to the category. The outer ring displays the individual metabolite IDs corresponding to each aroma category segment. Segment size is proportional to the number of metabolites in the category, and each aroma category is represented by a distinct color (consistent between the inner and outer rings).

Flavor attribute inference was conducted to link metabolite differences to flavor perception, based on database matching of differential metabolites to odor descriptors (Table S2) and selection of the top 10 core flavor categories by annotated metabolite count (Figure 7 and Figure S2) [6]. The results revealed distinct inferred flavor profiles for SH363 and SH361, with both varieties containing metabolites corresponding to all core categories and multiple extended descriptors, but significant differences in inferred intensity (Fisher’s exact test, *p* <0.05).

Key differences in inferred flavor characteristics included: SH363 exhibited a substantially higher number of metabolites associated with core categories “green” (5 metabolites: KMW0193119, XMW0004119, D371, KMW0129, KMW0604), “fresh” (4 metabolites: KMW0148011, NMW0783011, XMW0004119, D371), “pine” (4 metabolites: KMW0148011, KMW0193119, NMW0783011, XMW0004119), “woody” (4 metabolites: KMW0148011, KMW0193119, XMW0004119, KMW0604), and “resinous” (3 metabolites: KMW0193119, NMW0783011, XMW0004119) (Figure 7). These metabolites were predominantly terpenoids (e.g., β-pinene, KMW0193119; costunolide, Hmcp004865) and lipid-derived volatiles (e.g., 1-linoleoylglycerol, Lhmp112042) (Table S1). Notably, the same metabolites annotated to core categories “coffee-like” and “caramel-like” (2 metabolites each: KMW0132, XMW0651) were also matched to extended descriptor “roasted” in Table S2, leading to the inference of an overall roast-like aroma. Additionally, extended descriptors “aromatic” and “nutty” (Table S2) were associated with these metabolites, collectively contributing to a subtle nutty impression and multi-dimensional flavor profile. SH361 was characterized by a higher number of metabolites associated with core category “sweet” (2 metabolites: KMW0148*011, KMW0129) and significantly fewer annotated metabolites in all other core categories and extended descriptors (*p* < 0.05). This sugar-centric composition infers a predominantly sweet, simple flavor profile, lacking the aromatic depth of SH363.

Consistently, these inferred sensory differences align with metabolic chemistry: SH363 had a greater abundance of lipid-derived volatile compounds (annotated to “nutty” “roasted” in Table S2) and higher terpenoid content (contributing core category “green” “pine” notes), whereas SH361 contained higher sugar levels (annotated to “sweet” in core categories, generating little aroma on their own).

## 4. Discussion

### 4.1. Key Metabolites Affecting Sunflower Quality

The metabolic comparison between SH361 and SH363 provides a framework for understanding their quality differences. Flavonoids emerged as a prominent group among the differential metabolites, which is noteworthy because flavonoids have diverse bioactivities in plants [42]. For instance, quercetin and related flavonols are potent antioxidants that protect seeds from oxidative damage and prolong storage life [43]. Changes in flavonoid content can directly influence seed flavor: certain flavonoid glycosides impart bitterness or astringency, so their differential accumulation could alter taste perception [44]. Moreover, many flavonoids are involved in plant defense. The presence of specific flavonoids in higher amounts in SH363 may indicate an enhanced protective metabolism that also incidentally improves flavor (e.g., by reducing lipid oxidation and thus preventing rancidity or off-flavors). In particular, quercetin glycosides enriched in SH363 could inhibit lipid oxidation in the seeds [37], thereby suppressing the formation of rancid off-flavor compounds and helping preserve a fresh nutty taste.

Terpenoids are another major class of differential metabolites that likely drive aroma differences between the two varieties. Terpenes and terpenoids constitute key aroma compounds in many plants; for example, monoterpenes like geraniol and limonene impart fresh, floral notes, whereas sesquiterpenes such as farnesene contribute to more complex, earthy aromas [45,46]. In our data, SH363 had higher levels of certain terpenoids, which could underlie its stronger “nutty” and aromatic flavor profile. Terpenoids also serve as signaling molecules in plants, and their differential levels might reflect broader metabolic shifts influencing seed development and quality. It is plausible that SH363’s metabolic profile channels more precursors into terpene biosynthesis, thereby enhancing aroma-active compounds.

Besides flavonoids and terpenoids, other metabolite classes differed between SH363 and SH361 as well, including amino acids, lignans, and coumarins. Amino acids not only form proteins but also contribute to taste (e.g., glutamic acid and aspartic acid impart umami flavor) and serve as precursors in various metabolic pathways [47]. In our study, variations in amino acid levels might affect subtle savory or “brothy” taste notes. For instance, differences in an umami-related amino acids like glutamate between the varieties could influence the perceived savory aspect of their flavor. Lignans and coumarins, on the other hand, are related to seed structural components and defense mechanisms. Their differing levels may influence seed attributes such as texture (hardness or astringency) and indirectly affect flavor stability during storage (through antioxidant activity), although they are less directly tied to flavor perception compared to the major metabolite groups.

### 4.2. Metabolic Pathways Associated with Quality Differences

Pathway analysis reinforces the importance of secondary metabolism in the quality traits of these sunflower varieties. The “biosynthesis of secondary metabolites” pathway was highly populated with differential metabolites, underlining that flavor and aroma differences stem from broad changes across secondary metabolic networks [48]. For example, within the flavonoid biosynthesis sub-pathway, differences in enzyme activities (e.g., chalcone synthase, flavonol synthase) could lead to the flavonoid content variations we observed. Such enzymatic differences might be genetic or regulatory, suggesting potential targets for breeding or biotechnological intervention to enhance seed flavor (by boosting beneficial flavonoids or reducing bitter ones) [49,50].

Furthermore, the metabolic pathways for terpenoid and phenylpropanoid (flavonoid) biosynthesis are interconnected and draw from common primary precursors. The terpenoid precursor isopentenyl pyrophosphate (IPP) is produced via the mevalonate and MEP pathways drawing on acetyl-CoA and glyceraldehyde-3-phosphate, while flavonoid biosynthesis relies on aromatic precursors from the shikimate pathway [43]. This indicates a potentially shared carbon flux: carbon diverted towards IPP and terpenoid production could affect the availability of precursors for the shikimate-derived phenylpropanoid pathway, and vice versa. Such interconnections might allow SH363 to efficiently channel carbon into both pathways simultaneously, explaining how it achieves high levels of both terpenoids and flavonoids without the trade-offs that might be expected. In practical terms, this insight suggests that improving one class of metabolites (e.g., increasing terpenoids for enhanced aroma) could be balanced with another class (e.g., maintaining flavonoid levels for stability or taste) by managing the metabolic flux through these pathways.

The pentose and glucuronate interconversion pathway (part of carbohydrate metabolism), which provides NADPH and various sugar intermediates, was also significantly enriched in differential metabolites. We found that SH361, more so than SH363, accumulated higher levels of certain sugar alcohols (e.g., xylitol, D-arabinitol, ribitol) in this pathway, indicating an increased carbon flux through pentose-phosphate-related metabolism in SH361. This finding aligns with SH361’s higher soluble sugar content and suggests that SH361 directs more metabolic resources toward carbohydrate synthesis/storage. An enhanced flux through this pathway could increase NADPH availability for biosynthetic processes [51]. However, despite this carbohydrate-oriented metabolic strategy, SH363 demonstrated better flavor and nutritional metrics. This contrast implies that factors other than pentose-pathway activity (such as increased lipid and aroma compound synthesis in SH363) are key to its quality advantage. Additionally, the accumulation of sugar alcohols in SH361 may serve osmoprotective or storage functions, reflecting a distinct metabolic strategy for stress tolerance or growth that does not necessarily improve flavor.

### 4.3. Metabolite—Sensory Relationships

The flavor perception differences between SH361 and SH363 correlate closely with their metabolomic profiles. SH363’s superior flavor—often described as nuttier and more aromatic—is linked with its metabolic profile: higher terpenoid levels likely contribute to stronger “green,” “pine,” and resinous aroma notes, and certain flavonoids may yield unique aromatic compounds upon degradation [47]. Sweetness perception aligns with sugar content: SH361 has a substantially higher sugar level and indeed was noted (from prior descriptions) as tasting sweeter, though its flavor was somewhat one-dimensional. Bitterness or astringency can be influenced by specific flavonoids or alkaloids; SH361’s higher levels of some bitter-associated metabolites (for example, higher accumulation of certain phenolic compounds) might have contributed to its less favorable taste. Meanwhile, SH363’s reduced burden of bitter compounds and its relatively higher lipid content (neutral in taste and capable of smoothing flavor profiles) likely make it more palatable.

Linking specific metabolites to sensory attributes provides actionable insights for improving seed flavor. For example, both SH361 and SH363 exhibit “green” and “pine” aroma notes (likely due to shared terpene compounds such as hexenal or α-pinene); however, these notes are markedly more pronounced in SH363, possibly due to its higher total terpene abundance. Conversely, the absence or reduction in certain bitter compounds in SH363 (e.g., lower levels of bitter flavonoid glycosides or caffeoylquinic acids like chlorogenic acid) might contribute to its cleaner, more pleasant taste. Overall, these metabolite–sensory relationships point to specific compounds or classes that breeders could target to alter flavor: for instance, increasing terpenoids and sweet amino acids (such as alanine and glycine) while decreasing bitter flavonoids could plausibly enhance consumer appeal. We emphasize that these are hypotheses based on known flavor chemistry and our observed trends, rather than direct causal conclusions.

While our study demonstrates clear qualitative associations between metabolite profiles and sensory attributes, the limited sample scope (two varieties in one environment) precluded any robust statistical correlation analysis between individual metabolite concentrations and sensory intensity scores. Thus, our linkage of specific metabolites to sensory notes is based on established flavor chemistry knowledge and observed directional trends, rather than on direct statistical correlations from our data. We have made this distinction explicit in the text. Future studies that include more genotypes or larger sensory panels (with quantitative flavor ratings) could validate these proposed metabolite–sensory relationships—for example, by calculating correlation coefficients across diverse samples or using multivariate predictive models to link metabolite levels with sensory ratings.

### 4.4. Macronutrient Composition and Flavor

Our compositional analysis suggests that the stark difference in sugar and fat content between SH361 and SH363 plays a pivotal role in their flavor contrast. SH363’s seeds are rich in lipids and comparatively low in sugars, whereas SH361’s seeds show the opposite pattern. This imbalance in macronutrients likely underlies SH363’s pronounced nutty flavor versus SH361’s relatively flat, overly sweet taste. Lipids contribute to flavor in multiple ways: they serve as precursors for aromatic compounds generated during processes like roasting, and they dissolve and stabilize many volatile flavor molecules, aiding their retention in the food matrix. Lipids also provide a desirable mouthfeel and can carry lipid-soluble aroma compounds. In SH363, the ample fat content (along with sufficient amino acids and other precursors) enables the formation of a complex, savory flavor profile with roasted-nutty notes. By contrast, SH361’s high sugar content, without a commensurate level of fat, leads to an overly sweet, one-dimensional flavor profile.

It is true that elevated sugar levels can enhance certain Maillard reaction-derived flavors up to a point [7]. However, in SH361 the excess sugars likely led to a dominance of sweetness that was not balanced by other flavor notes. Meanwhile, SH363’s high oil content (typical of confectionery sunflower seeds) facilitated the production of the rich, nutty aroma that consumers prefer [52]. Fats also carry natural antioxidants and can suppress off-flavors; therefore, the low fat in SH361 could mean not only fewer lipid-derived aromatic compounds but also less “buffering” of undesirable tastes (such as burnt or bitter notes). Essentially, SH363’s lipid-rich composition contributes to a fuller, rounded flavor, whereas SH361’s sugar-rich composition lacks depth. This finding underscores that optimizing the sugar-to-fat ratio in sunflower seeds is critical for achieving high flavor quality.

Moreover, lipid oxidation products contribute importantly to roasted flavor chemistry. As seeds are heated (e.g., during roasting), oxidation of unsaturated fatty acids produces aldehydes such as hexanal. These aldehydes can participate in Strecker degradation reactions with amino acids to form heterocyclic compounds like pyrazines [7,53]. Pyrazines are well-known for imparting strong roasted, nutty aromas. This lipid-driven Strecker pathway likely enhances the nutty, savory notes in the high-oil SH363. In contrast, SH361’s lower lipid content would limit this flavor-generating mechanism. Thus, the synergy between lipids and Maillard-type reactions (mediated via Strecker aldehydes and subsequent pyrazine formation) provides a biochemical explanation for why a higher oil content is associated with a more pronounced roasted-nutty flavor in sunflower seeds.

Notably, SH363’s macronutrient profile (lower sugar, higher fat) aligns with classic confectionery sunflower seed varieties known for superior nutty flavor, whereas SH361’s atypically high sugar content may have been selected for agronomic traits like yield or large seed size at the expense of flavor [6]. The superior flavor and market value of SH363 attest to the importance of macronutrient composition in driving consumer preferences. Breeding programs may thus consider selecting for higher seed oil content (to a point that does not compromise other agronomic traits) and moderate sugar levels in order to improve the flavor of sunflower seeds.

### 4.5. Study Limitations and Future Prospects

While this study provides a comprehensive metabolomic and sensory profile analysis of two sunflower varieties, some limitations should be acknowledged. First, we focused exclusively on mature seeds from a single harvest; metabolite profiles can change during seed development and post-harvest storage. Future studies should examine multiple developmental stages (especially key phases like seed filling and oil accumulation) and storage periods to capture how metabolic changes over time affect flavor and quality formation. Second, we concentrated on metabolite differences, but underlying gene expression and enzyme activity differences likely drive those metabolic changes. Integrating transcriptomic and proteomic data in the future would provide a deeper understanding of the regulatory mechanisms governing these quality traits. Third, environmental factors (soil, climate) were controlled and kept constant in this study (a single location and season), but in broader cultivation these factors can significantly influence metabolite levels and flavor. Investigating how different growing environments (locations, climates, agronomic conditions) affect the sunflower seed metabolome will be important to determine the robustness of these flavor-associated metabolic traits and to guide multi-environment selection strategies in breeding.

Furthermore, our analysis provides only a static snapshot of the metabolome under a specific set of conditions. In reality, metabolite levels and flavor profiles are dynamic and influenced by both developmental timing and environmental interactions. It is a limitation of our work that we did not capture the dynamic changes in metabolism during seed development or under varying environmental scenarios. In future research, developmental time-course metabolomic analyses (e.g., sampling seeds at early, mid, and late maturation stages) would clarify when critical flavor- and nutrition-related metabolites accumulate. For instance, monitoring the oil biosynthesis phase could reveal how and when flavor precursors (like terpenoids or Strecker aldehydes) peak. Additionally, conducting trials across multiple environments (different locations, climates, or soil conditions) will be essential to validate whether the metabolic and sensory differences observed between SH363 and SH361 hold true under diverse growing conditions. Such multi-environment data would help ensure that any breeding strategies based on our findings are robust and effective across the range of environments in which sunflower is cultivated.

Despite these limitations, this study provides valuable clues for sunflower quality improvement. In the future, through the integrated application of multi-omics technologies, in-depth studies on environmental effects, and the use of genetic engineering and advanced breeding techniques, we expect to further unravel the complex mechanisms of sunflower seed quality formation and develop sunflower varieties with truly superior flavor and nutritional profiles. We also acknowledge that we did not measure certain physicochemical parameters (such as peroxide value, acid value for lipid oxidation stability, moisture content, or perform a targeted volatile aroma analysis using GC–olfactometry) in this study. Including these standard quality assays in future work would further strengthen the causal links between metabolic differences and sensory outcomes, particularly regarding lipid oxidation and aroma development, and provide a more robust, quantitative basis for our proposed flavor mechanisms.

## 5. Conclusions

In this study, a comprehensive metabolomic analysis of sunflower varieties SH361 and SH363 was performed. A total of 1448 metabolites were detected, and 242 differential metabolites were identified between the two varieties, distributed mainly among flavonoids, terpenoids, and other secondary metabolite categories. These differential metabolites were significantly enriched in pathways related to secondary metabolite biosynthesis and carbohydrate metabolism. Notably, although SH361 exhibited elevated levels of certain pentose-derived sugar alcohols (consistent with its higher sugar content), SH363 still demonstrated superior flavor and nutritional quality. This suggests that in SH363, carbon flux is preferentially directed towards lipids and volatile flavor compound production, underpinning its quality advantage. This finding supports a working model of lipid-associated flavor enhancement in sunflower seeds: higher lipid accumulation could lead to greater generation and retention of aroma compounds, thereby potentially amplifying flavor complexity.

Overall, our results provide a detailed understanding of the metabolic basis of quality differences between SH361 and SH363. We offer both practical information and theoretical insights for sunflower quality improvement and breeding programs. In particular, the concept of lipid-associated flavor enhancement and the identification of key flavor-related metabolic pathways enable breeders to target specific metabolites and pathways in developing new sunflower varieties with superior taste, aroma, and nutritional value. For example, breeding strategies might focus on upregulating terpenoid biosynthesis (to boost aroma-rich terpene levels) and optimizing the seed sugar–lipid ratio (to balance sweetness and nuttiness) as a means of enhancing flavor. By leveraging such metabolic targets, it becomes possible to design sunflower cultivars that are both high-yielding and flavor-rich. In essence, our study presents a metabolic correlation model linking sunflower seed metabolites with sensory quality, and lays a foundation for flavor-directed sunflower breeding–where key pathways (e.g., lipid accumulation, terpenoid biosynthesis) are engineered or selected to produce sunflower seeds with improved flavor without sacrificing agronomic performance. Breeders may also utilize the identified flavor-associated metabolites as molecular or biochemical markers in selection programs (for example, via metabolomics-assisted or marker-assisted breeding) to screen and develop superior-tasting sunflower lines. Future research should build on these findings to validate the proposed flavor mechanisms and further refine these breeding interventions, ultimately paving the way for sunflower varieties that delight consumers with their taste while maintaining high yield and nutritional value.

## Figures and Tables

**Table 1:** Nutritional composition of SH361 and SH363 sunflower seeds (mean ± SD, *n* = 3), expressed on a dry-weight basis. Values in bold indicate a significant difference between varieties (two-sample t-test, *p* < 0.05).

Parameter (% Dry Weight)	SH361 (Mean ± SD)	SH363 (Mean ± SD)
Total sugars	**31.96 ± 1.91**	**24.62 ± 2.65**
Crude fat	**23.77 ± 0.06**	**51.01 ± 0.54**
Crude protein	26.96 ± 0.32	26.08 ± 0.39
Starch	2.00 ± 1.4	2.00 ± 0.3
Cellulose	3.37 ± 3.97	3.57 ± 3.98

## Data Availability

The original contributions presented in this study are included in the article/Supplementary Materials. Further inquiries can be directed to the corresponding authors.

## Supplementary Materials

The following supporting information can be downloaded at: https://www.mdpi.com/article/10.3390/foods15010106/s1, Figure S1: EI-SIM total ion chromatogram of a sunflower seed sample; Figure S2: Sankey diagram mapping specific differential metabolites to aroma descriptors; Table S1: Differential metabolites between SH363 and SH361(SH363 vs. SH361); Table S2: Significant metabolite flavor characteristics differences between SH363 and SH361.
