# Supplementary material for: Integrated Metabolomics and Flavor Profiling Provide Insights into the Metabolic Basis of Flavor and Nutritional Composition Differences Between Sunflower Varieties SH363 and SH361
Source: Foods. 2025 Dec 30;15(1):106. doi: 10.3390/foods15010106 (PMC12785768; doi:10.3390/foods15010106)
Supplement: Supplementary file 1 [file foods-15-00106-s001.zip › Figure Supplements.pdf]

Integrated Metabolomics and Flavor Profiling Provide Insights into the Metabolic Basis of Flavor and Nutritional Composition Differences Between Sunflower Varieties SH363 and SH361

Yanli Li[1], Huihui Gong[1], Xinxiao Cui[1], Xin Wang[1], Ying Chen[1], Huiying Li[2,]*, Junsheng Zhao[1,]*

[1] Institute of Industrial Crops, Shandong Academy of Agricultural Sciences, Jinan 250100, Shandong, China.

[2] Sunflower Research Institute, Baicheng Academy of Agricultural Sciences, Baicheng 137000, Jilin, China.

*Correspondence: Junsheng Zhao (zhaojunsheng@saas.ac.cn)

Huiying Li (leahuiy@163.com)

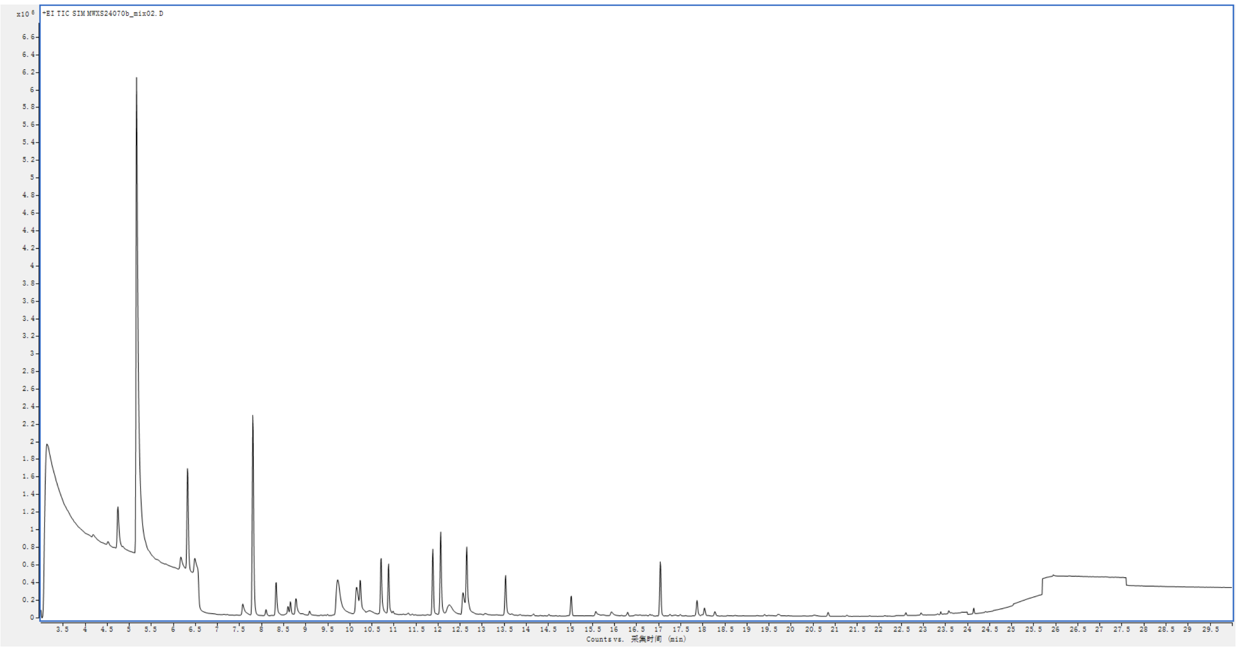

Figure S1. EI-SIM total ion chromatogram of a sunflower seed sample**.** The total ion chromatogram (TIC) of a sunflower seed sample acquired in electron ionization-selected ion monitoring (EI-SIM) mode: the x-axis represents retention time, the y-axis denotes ion counts, and the peaks correspond to the relative abundances of different metabolites. This plot serves as supplementary quality control data for the metabolomic analysis of the sample.

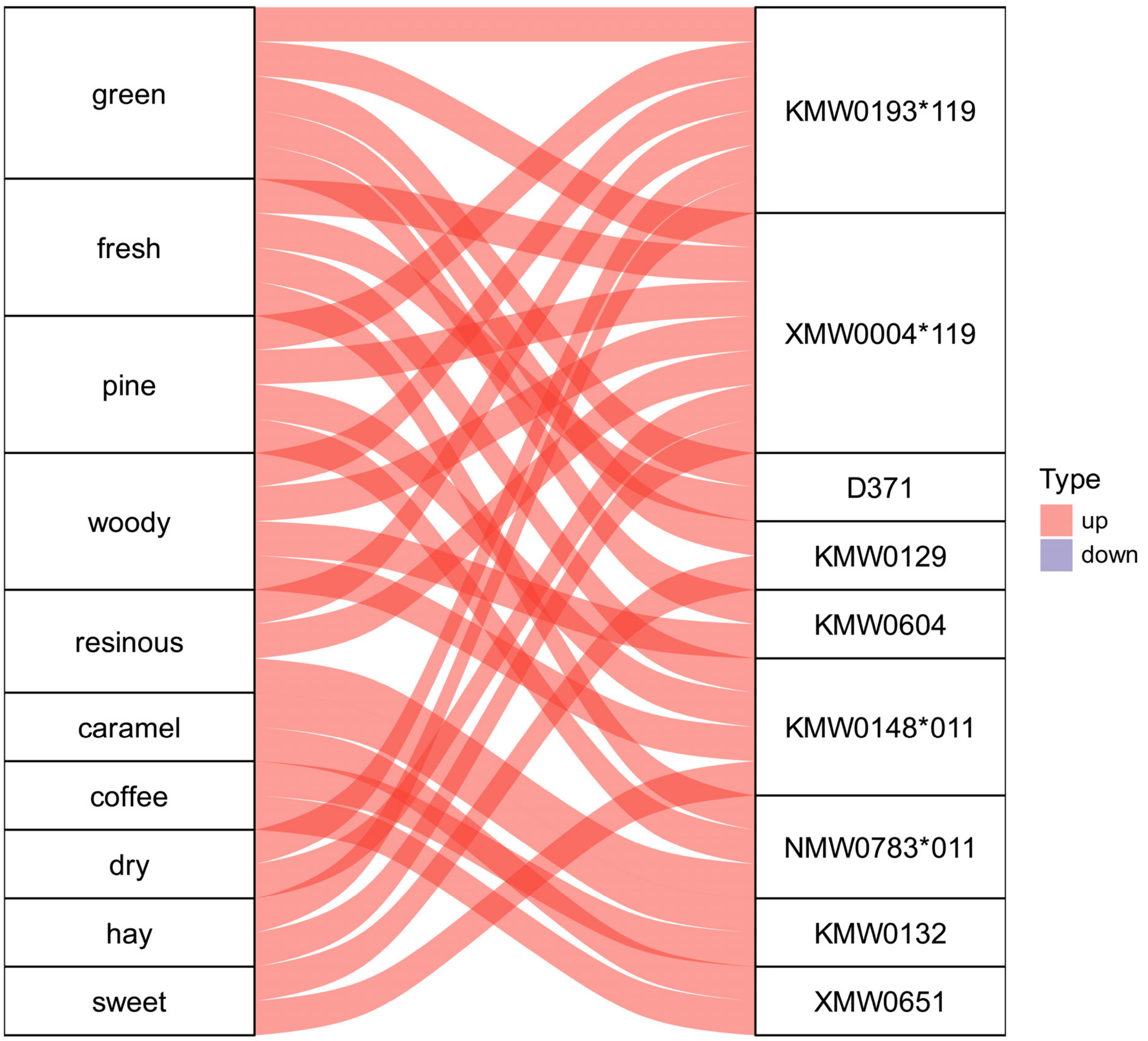

Figure S2. Sankey diagram mapping specific differential metabolites to aroma descriptors. Left-side nodes are aroma descriptor categories, and right-side nodes are metabolite identifiers. Flows connect metabolites to the aroma categories they influence. Flow width is proportional to the relative concentration difference for each metabolite, and flows are color-coded by direction of change (red: higher in SH363; blue: higher in SH361). Only significantly altered aroma-associated metabolites are shown.
